# Annotations

**Published:** 1891-10-03

**Authors:** 


### Annotations.

**African Philanthropy and Dividends.**

The Hospital has always maintained the policy of developing mankind on the principle of a sound mind in a sound body. As man is a moral and intellectual as well as a material animal, a true science of medicine must inevitably take cognisance of mind and soul as well as of body. Moreover, as Carlyle said, "All men make up mankind," and the individual man is never so well understood as when he is seen through the mirror of the race. Hence we are staunch believers in a widespread British Empire, and in the extension of our benevolent and reasonable religion in all possible directions. Africa, the dark continent, needs the just and sound-hearted Englishman almost more than any other part of the world. She needs his religion and she needs his trade. Missionary societies are great civilisers. We know this of our own personal knowledge; and therefore we are absolutely indifferent to the feeble objections of the sceptical cynic. Trade, too, is an almost unmixed blessing to native African races. Those races have practically everything to gain and nothing to lose by the opening out of their country to British trade. For the benefit of our readers who do not know the facts, we may briefly state that there is a British East Africa Company, with Sir Francis de Winton as its inspiring spirit, Sir William Mackinnon as its active trading head, and Captain Lugard as its executive leader. The object of this company is to make a state under British protection of the Uganda country, with the full concurrence of the King and the people; and to carry a railway from the coast right through the country up to the lake on its distant side. This would open out the country both to Christian missions and to British trade. There is not now, however, and there cannot be for a long time, any such measure of trade as will repay outlay. The company, as it is said, must "take out its dividends in philanthropy." Now there is no doubt whatever of two things: first, that the making of the proposed railway from the coast to the lake, and the administration of the country under English control would be a blessing to the native populations which it is impossible to over-estimate. Moreover, the native populations ardently wish for it. Secondly, the leaders of the expedition, Sir Francis de Winton, Sir William Mackinnon, and Captain Lugard, are men of great business capacity and of the highest personal character. Of Captain Lugard a lady who has lived ten years in the Uganda country, and who knows him intimately, said to the present yesterday, that it was impossible for him to do anything wrong, or mean, or unkind. Well, but in spite of the necessity, the benevolent necessity, for this expedition, it is much feared that it will have to be withdawn; and withdrawn because it is proving to be so costly, that the philanthropic promoters are coming to the end of their resources. But this must be prevented: and that not in the interests of the promoters, but in the interests of the natives who are harried by slave-hunters; of the Christian religion, which is welcomed by the people on all hands, and of the honour of the British Empire, which has taken over the protectorate of the country under the recent treaty with Germany, and which cannot, therefore, without national dishonour, refuse to fulfil its just obligations. The question is one of money; but not of a large sum of money. It is not a question of armies at all, but of the mere bagatelle of forty thousand a year for a few preliminary years. If our Government would undertake to subsidize the British East Africa Company to the extent of forty thousand a year for the next five or six years, that is, if it would make itself responsible for the sum of less than a quarter of a million altogether, it would secure the whole Uganda country for expanding British enterprise, it would deliver that country from the terrors of the slave-hunter, it would connect the east coast of Africa with one of its largest central lakes by a railway, and it would bring all the people along the line of route and for hundreds of miles round the central lake, within the radius of civilising and Christianising influences. All this can be done for forty thousand a year. Irregular enterprise in Africa would thus be brought under control, and such ardent and effervescent spirits as Mr. H. H. Johnstone also would be bottled up until such times as they could be utilised by unagressive persons of proved discretion. The thing must be done. The subsidy must be made. Civilisation, Christianity, and British honour cannot be sacrificed for the sake of a miserable forty thousand a year.

**Animated Water at Birmingham.**

Birmingham is a most interesting town. Its history has shown, better than that of any other place of the same size, what the self-reliant Englishman is made of. It has dictated policies to the empire in past times, and it is still ambitious to play its ancient *rôle*. Alas! that a civic state with such a history should have one unhappy and widely-extending blot on its self-governing escutcheon! Birmingham cannot supply itself with pure water. There is no doubt whatever about the fact; we have our information both from public and private sources. It was announced in a Birmingham newspaper the other day that Councillor Barclay would deliver a lecture at the Midland Institute on "Our Future Water Supply." Concerning this innocent-looking announcement the newspaper in question grimly remarks: "If the gentleman who is announced to lecture on the Corporation water supply tonight had visited this office recently, he might have been supplied with a most interesting variety of subjects for his limelight views. He would thus be able to describe and illustrate not only the Corporation water supply, but also the Corporation aquarium, as it exists in reservoirs and many miles of street mains. Our readers are aware that on several occasions for months past we have described specimens of the live stock discovered in the water which had been brought under our notice, but if we had cared to store the many bottled specimens which have been sent to the office we should have acquired a wonderful collection. Another bottle of animated water reached us this morning. It is before us as we write, and in it can be counted at least a dozen aquatic insects, each as big as a fully-matured earwig, and very similar in appearance. Besides these pretty little creatures, this crystal liquid which people are supposed to drink with relish, contains hundreds of a species of small tape-worm which are engaged in a battle-royal with the gentry of the earwig extraction. The thought that these autumn naval manœuvres—so to speak—might be going on in your inside—and probably are—is by no means a pleasant one." The Birmingham writer is witty; it is lucky for him that he can be so. For our part, the knowledge that we were drinking water every day which was charged with the ova of the tape-worm would dispose us to other thoughts than those of wit. From a private correspondent we have the following: "I can vouch for the truth of the statements," about the water supply, "for I am compelled to have filters attached to all my water taps in addition to the table filters." This correspondent urges upon us very strongly the necessity of dealing with the whole question from an independent standpoint. "You will have the support of the doctors," he says, "for the conscientious ones are loud in their complaints, although they earn their living from the maladies and diseases created and disseminated by the water." After the water supply has been dealt with, our correspondent advises us to take up the "Drainage and Midden system," which, he says, is "horrible." "I could make your hair stand on end with experiences I have personally passed through" he adds. How are the mighty fallen, if this be true! Birmingham, which essays to lead the empire, cannot manage its own drains, and its own water supply. What is the reason? Is it want of brains? Or is it municipal parsimony? Where is Mr. Chamberlain? And where is Dr. R. W. Dale? Above all, where is Sir Walter Foster? For ourselves, we are astonished; we cannot believe the thing to be true. There the statements stand, however: what have the authorities to say for themselves?

